# Editorial: Compositional Diversity in Cereals in Relation to Their Nutritional Quality and Health Benefits

**DOI:** 10.3389/fnut.2021.819923

**Published:** 2022-01-07

**Authors:** Jinsong Bao, Lovemore Nkhata Malunga

**Affiliations:** ^1^Institute of Nuclear Agricultural Sciences, College of Agriculture and Biotechnology, Zhejiang University, Hangzhou, China; ^2^Department of Food and Human Nutritional Sciences, University of Manitoba, Winnipeg, MB, Canada

**Keywords:** cereals, genetic diversity, nutritional quality, end-use quality, health benefit

Continued population increase and climate change have given the food and agricultural industry new tasks to not only produce large quantities of food, but also with better nutritional quality and functionality. Yield achievements in many countries are very significant for cereals, however, the nutrition and grain quality improvement lag much behind. Characterization of the compositional diversity in cereals may provide important information for breeders and processors to explore the nutrient diversity. It is also well-known that phytochemical concentrations are not only affected by genetic background, but also by environments and processing techniques. This Research Topic is aimed at collecting papers suitable to improve our knowledge on the compositional diversity, the structural and functional relations of the phytochemicals in cereals highlighting the importance of diversity in the context to food chemistry, food safety, and human health.

In this special e-collection, seven papers covering the rice, maize, wheat, rye, barley, and oil seed crops were accepted for publication. The compositions investigated include volatile compounds in rice (Ashokkumar et al.), phenolic acids in maize (Zavala-López et al.) and barley spent grain (Rahman et al.), amino acids profile in wheat (Siddiqi et al.), rye, and barley (Rani et al.), fatty acids profile in oil seeds (Deme et al.), zinc concentration in wheat (Wang et al.). The total phenolic content and antioxidant activity were also investigated in rice (Ashokkumar et al.), barley spent grain (Rahman et al.), and oil seeds (Deme et al.).

Wheat is one of the most cultivated crops in the world and is the principal source of energy, protein, fiber, vitamins, and phytochemicals to nearly 2.5 billion people. The variation in the flour characteristics, protein profiling, proportion of the different protein fractions, and amino acid composition have been found in 14 wheat cultivars (Siddiqi et al.). Lysine is the most limiting amino acid in all wheat varieties which had the lowest amino acid score. Significant correlations between various grain and flour parameters are beneficial to plant breeders, millers, and bakers in selecting those wheat varieties with better quality characteristics (Siddiqi et al.). The technological, functional, and physicochemical properties of wheat, rye, and barley cultivars were compared (Rani et al.). The physiochemical parameters showed variability among the three cereal grains. Lactic acid-solvent retention capacity (SRC) was found to be higher in wheat than rye and barley, indicating higher gluten strength. Sucrose-SRC and sodium carbonate-SRC were higher in rye than wheat and barley flours. The essential amino acid proportion in barley and rye cultivars was higher than wheat cultivars. Barley and rye flours have higher biological value owing to their higher lysine content. The results would help manufacturers and researchers to develop healthy food products based on rye and barley flour (Rani et al.). Zinc (Zn) is an essential trace element for the growth and development of humans, animals, and plants. Insufficient Zn intake can cause loss of appetite, growth retardation, rough and peeling skin, and immune system dysfunction. Biofortification of zinc in wheat may be an effective way to solve human Zn deficiency. Wang et al. briefly reviewed the content, distribution, and bioavailability of zinc element in wheat grain, and wheat-derived foods. The review suggests that Zn deficiency can be addressed through breeding for high zinc wheat cultivars, and reducing anti-nutritional factors through wheat processing techniques.

Maize is a staple crop for developing countries especially in Latin American and large regions in Africa. The phenolic acids have proven to be associated with the improvement of human health because of their antioxidant capacity. Wide ranges in trans-ferulic acid, *p*-coumaric acid, and two types of diferulic acid were found in 20 landraces and 25 modern inbred lines hybrids, showing the diversity of the genotypes for both groups. The only phenolic compound able to detect a difference between modern hybrids and landraces hybrids is *p*-coumaric acid, in both its soluble and cell-wall bound forms. The effect of agro-ecological origin groups was significant for soluble *p*-coumaric and trans-ferulic acids, denoting the role of phenolic compounds in the ability of maize to endure different environmental conditions (Zavala-López et al.). The composition of phenolic acids and antioxidant activities in the defatted brewers spent grain meal (barley) were evaluated after they were subjected to various heat treatments. Heating leads to an increase in the total soluble phenolic content, total flavonoid content, and the antioxidant activities. These results confirm the ability of heat processing to release bioactive phenolic from their bound forms thereby enhancing the phenolic acids and the digestibility of brewers spent grain meal in the intestinal tract (Rahman et al.). Genotypic diversity in the total phenolic content, antioxidant activity, carotenoid, and chlorophyll contents was compared between five varieties of niger seed (*Guizotia abyssinic*a) and eight varieties of linseed (*Linum usitatissimum*). As both are oil crops, they showed major differences in several characteristics although both are rich in bioactive compounds. In niger seed oil, linoleic acid (C18:2) is the dominant fatty acid, accounting for 73.3–76.8% of the total fatty acids. In linseed oil, linolenic acid (C18:3) is the dominant fatty acid accounting for 55.7–60.1% (Deme et al.).

Rice (*Oryza sativa* L.) is one of the major cereal crops cultivated across the world, particularly in Southeast Asia with 95% of global production. Total phenolic content and the volatile organic compounds concentrations were compared between eight traditional and two modern rice varieties cultivated in South India (Ashokkumar et al.). Significant variations exist for fatty acids, total terpenes, total phenols, total aliphatic alcohols, total alkanes, and total alkenes among the rice varieties. Traditional varieties had higher variability for nutrients and aroma than modern rice, which can be used as potential parents to improve modern high-yielding varieties for the evolving nutritional market.

In summary, the results of the above mentioned studies and reviews represent a large amount of new relevant data on the compositional diversity in cereals. Phenolic acids and antioxidant activities are among the trending topics in cereal research as consumption of diets high in phenolic compounds are perceived to contribute positively to human health. The research topic has reinforced the notion that nutrient and phytochemical composition varies with genotype, growing environment, and processing techniques. Hopefully, the readers can find the papers interesting and informative enough to their research.

## Author Contributions

JB wrote the editorial. LNM reviewed the editorial. Both authors contributed to the article and approved the submitted version.

## Conflict of Interest

The authors declare that the research was conducted in the absence of any commercial or financial relationships that could be construed as a potential conflict of interest.

## Publisher's Note

All claims expressed in this article are solely those of the authors and do not necessarily represent those of their affiliated organizations, or those of the publisher, the editors and the reviewers. Any product that may be evaluated in this article, or claim that may be made by its manufacturer, is not guaranteed or endorsed by the publisher.

